# Efficacy and safety of CPX-351 versus 7 + 3 chemotherapy by European LeukemiaNet 2017 risk subgroups in older adults with newly diagnosed, high-risk/secondary AML: post hoc analysis of a randomized, phase 3 trial

**DOI:** 10.1186/s13045-022-01361-w

**Published:** 2022-10-26

**Authors:** Jorge E. Cortes, Tara L. Lin, Kobby Asubonteng, Stefan Faderl, Jeffrey E. Lancet, Thomas Prebet

**Affiliations:** 1 Department of Leukemia, University of Texas MD Anderson Cancer Center, Houston, TX USA; 2 Georgia Cancer Center, Augusta University, 1410 Laney Walker Rd, CN2222, Augusta, GA 30912 USA; 3 Division of Hematologic Malignancies and Cellular Therapeutics, University of Kansas Medical Center, Kansas City, KS USA; 4 Department of Biostatistics, Jazz Pharmaceuticals, Philadelphia, PA USA; 5 Department of Clinical Development, Jazz Pharmaceuticals, Palo Alto, CA USA; 6 Department of Malignant Hematology, H. Lee Moffitt Cancer Center & Research Institute, Tampa, FL USA; 7 Department of Hematology, Yale School of Medicine, New Haven, CT USA

**Keywords:** Acute myeloid leukemia, Chemotherapy, CPX-351, European LeukemiaNet 2017 risk subgroup, Post hoc

## Abstract

CPX-351 (Europe: Vyxeos^®^ liposomal; United States: Vyxeos^®^) is a dual-drug liposomal encapsulation of daunorubicin and cytarabine in a synergistic 1:5 molar ratio. In a phase 3 study in older adults with newly diagnosed, high-risk/secondary AML, CPX-351 improved the remission frequency, overall survival, and post-transplant survival versus 7 + 3. This post hoc analysis evaluated the final 5-year follow-up outcomes according to the European LeukemiaNet 2017 risk classification. CPX-351–treated patients had a higher remission frequency (adverse risk: 41% vs 26%; intermediate risk: 58% vs 39%) and longer median overall survival (adverse risk: 7.59 vs 5.52 months; intermediate risk: 11.86 vs 7.75 months) and post-transplant survival (adverse risk: 43.14 vs 7.08 months; intermediate risk: not reached vs 13.57 months) versus 7 + 3, with outcomes generally poorer among patients with adverse-risk AML. The safety profile of CPX-351 among patients with adverse-risk or intermediate-risk AML was consistent with that of the overall study population. Early mortality was lower, and hospitalization length of stay per patient-year was shorter with CPX-351 versus 7 + 3 within the adverse-risk and intermediate-risk subgroups. The favorable outcomes observed with CPX-351 in this post hoc analysis are consistent with results for the overall study population and further support the use of CPX-351 in these patients.

ClinicalTrials.gov Identifier: NCT01696084.

**Supplementary Information:**

The online version contains supplementary material available at 10.1186/s13045-022-01361-w.

CPX-351 (Europe: Vyxeos® liposomal; United States: Vyxeos®) is a dual-drug liposomal encapsulation of daunorubicin and cytarabine in a synergistic 1:5 molar ratio [1]. Approvals of CPX-351 for newly diagnosed, therapy-related acute myeloid leukemia (AML) or AML with myelodysplasia-related changes (AML-MRC) in Europe (adults) and the United States (patients aged ≥ 1 year) [2, 3] were based on the results of a randomized, phase 3 study that demonstrated improved overall survival (OS), remission, and post-hematopoietic cell transplantation (HCT) survival with CPX-351 versus conventional 7 + 3 chemotherapy, with a similar safety profile [4].

The phase 3 study of CPX-351 versus 7 + 3 prospectively evaluated patients’ prognostic risk per National Comprehensive Cancer Network (NCCN) criteria, as the study was conducted in North America. However, in 2017, the European LeukemiaNet (ELN) provided updated recommendations on the diagnosis and management of adults with AML, including criteria for patient stratification into prognostic risk groups based on cytogenetic and molecular characteristics, which may inform treatment decisions [5]. Given differences between the ELN and NCCN risk criteria and the broad use of the ELN classification in clinical practice, this post hoc analysis evaluated long-term outcomes with CPX-351 versus 7 + 3 among subgroups of enrolled patients reclassified according to the ELN 2017 classification.

The design and methods of this randomized, open-label, phase 3 study (ClinicalTrials.gov Identifier: NCT01696084) were described previously [4]. Patients aged 60 to 75 years with newly diagnosed, high-risk/secondary AML were randomized 1:1 to receive up to 2 induction cycles of CPX-351 (100 units/m^2^ via 90-min infusion on Days 1, 3, and 5; second induction: Days 1 and 3) or 7 + 3 (cytarabine 100 mg/m^2^/day continuous 7-day infusion plus daunorubicin 60 mg/m^2^ on Days 1–3; second induction: 5 + 2 schedule) followed by up to 2 post-remission consolidation cycles with CPX-351 (65 units/m^2^) or 5 + 2. Patients were followed up for 5 years or until death. In this post hoc analysis, patients were reclassified into ELN 2017 risk subgroups based on their baseline characteristics [5]. The distribution of time-to-event endpoints was estimated using the Kaplan-Meier method, with hazard ratios and 95% confidence intervals (CIs) estimated using a Cox proportional hazards regression model stratified by age and AML subtype.

The study protocol and all amendments were approved by the institutional review board/ethics committee at each site. All patients provided written informed consent prior to study participation.

Results for the overall study population were described previously [4, 6]. Of 309 randomized patients, 297 (96%) had baseline characteristics permitting reclassification per the ELN 2017 risk criteria (Table 1). The majority (67%) of patients had adverse-risk AML; of these, *TP53* mutations were detected for 24% and 31% of patients in the CPX-351 and 7 + 3 arms, respectively. Most patients in both arms had an Eastern Cooperative Oncology Group performance status of 0–1. Only 6% of patients had favorable-risk AML (CPX-351: *n* = 10; 7 + 3: *n* = 7); within this subgroup, 9/10 (90%) and 6/7 (86%), respectively, achieved complete remission (CR) or CR with incomplete neutrophil or platelet recovery (CRi), and 6/10 (60%) and 5/7 (71%) had died at the time of this analysis.

**Table 1:** Baseline characteristics by ELN 2017 risk subgroup

	Intermediate-risk AML		Adverse-risk AML	
	CPX-351 (*n* = 40)	7 + 3 (*n* = 41)	CPX-351 (*n* = 99)	7 + 3 (*n* = 100)
Median age (range), years	69 (61, 75)	68 (60, 75)	68 (60, 75)	68 (60, 75)
Age subgroup, *n* (%)				
60 to 69 years	25 (63)	26 (63)	62 (63)	65 (65)
70 to 75 years	15 (38)	15 (37)	37 (37)	35 (35)
Male, *n* (%)	29 (73)	25 (61)	57 (58)	63 (63)
ECOG performance status, *n* (%)				
0	11 (28)	14 (34)	20 (20)	29 (29)
1	25 (63)	23 (56)	70 (71)	57 (57)
2	4 (10)	4 (10)	9 (9)	14 (14)
AML subtype, *n* (%)				
t-AML	9 (23)	3 (7)	17 (17)	27 (27)
AML with antecedent MDS				
With prior HMAs	19 (48)	19 (46)	29 (29)	32 (32)
Without prior HMAs	6 (15)	12 (29)	12 (12)	6 (6)
AML with antecedent CMML	2 (5)	4 (10)	7 (7)	6 (6)
de novo AML with MDS karyotype	4 (10)	3 (7)	34 (34)	29 (29)
NCCN cytogenetic risk, *n* (%)				
* n*	39	37	92	97
Better risk	0	1 (3)	0	0
Intermediate risk	31 (79)	31 (84)	30 (33)	26 (27)
Poor risk	8 (21)	5 (14)	62 (67)	71 (73)
*TP53* mutation, *n* (%)	–	–	24 (24)	31 (31)
WBC count, *n* (%)				
* n*	40	40	99	100
< 20,000/µL	33 (83)	35 (88)	87 (88)	83 (83)
≥ 20,000/µL	7 (18)	5 (13)	12 (12)	17 (17)
Platelet count, *n* (%)				
* n*	40	40	99	99
≤ 50,000/µL	20 (50)	21 (53)	66 (67)	62 (63)
> 50,000/µL	20 (50)	19 (48)	33 (33)	37 (37)
Median bone marrow blasts (range)^a^, %	36 (5, 80)	35 (6, 88)	35 (5, 93)	35 (3, 97)
Bone marrow blasts, *n* (%)				
* n*	38	40	97	97
< 20%	5 (13)	4 (10)	15 (15)	18 (19)
20% to 40%	14 (37)	19 (48)	42 (43)	41 (42)
> 40% to 60%	10 (26)	10 (25)	20 (21)	18 (19)
> 60%	9 (24)	7 (18)	20 (21)	20 (21)

*AML* acute myeloid leukemia; *CMML* chronic myelomonocytic leukemia; *ECOG* Eastern Cooperative Oncology Group; *ELN* European LeukemiaNet; *HMA* hypomethylating agent; *MDS* myelodysplastic syndrome; *NCCN* National Comprehensive Cancer Network; *t-AML* therapy-related acute myeloid leukemia; *WBC* white blood cell

^a^Data available for 34 and 37 patients in the CPX-351 and 7 + 3 arms, respectively, within the intermediate-risk subgroup and 93 and 91 patients within the adverse-risk subgroup

Remission occurred more frequently with CPX-351 versus 7 + 3 among patients with intermediate-risk (CR + CRi: 58% vs 39%) and adverse-risk AML (41% vs 26%; Fig. 1A). Among patients with adverse-risk AML, the remission occurred more frequently with CPX-351 versus 7 + 3 among those without *TP53* mutations (CR + CRi: 33/75 [44%] vs 15/69 [22%]) but was similar among those with *TP53* mutations (8/24 [33%] vs 11/31 [35%]; Additional file 1: Table S1).

**Fig. 1:**
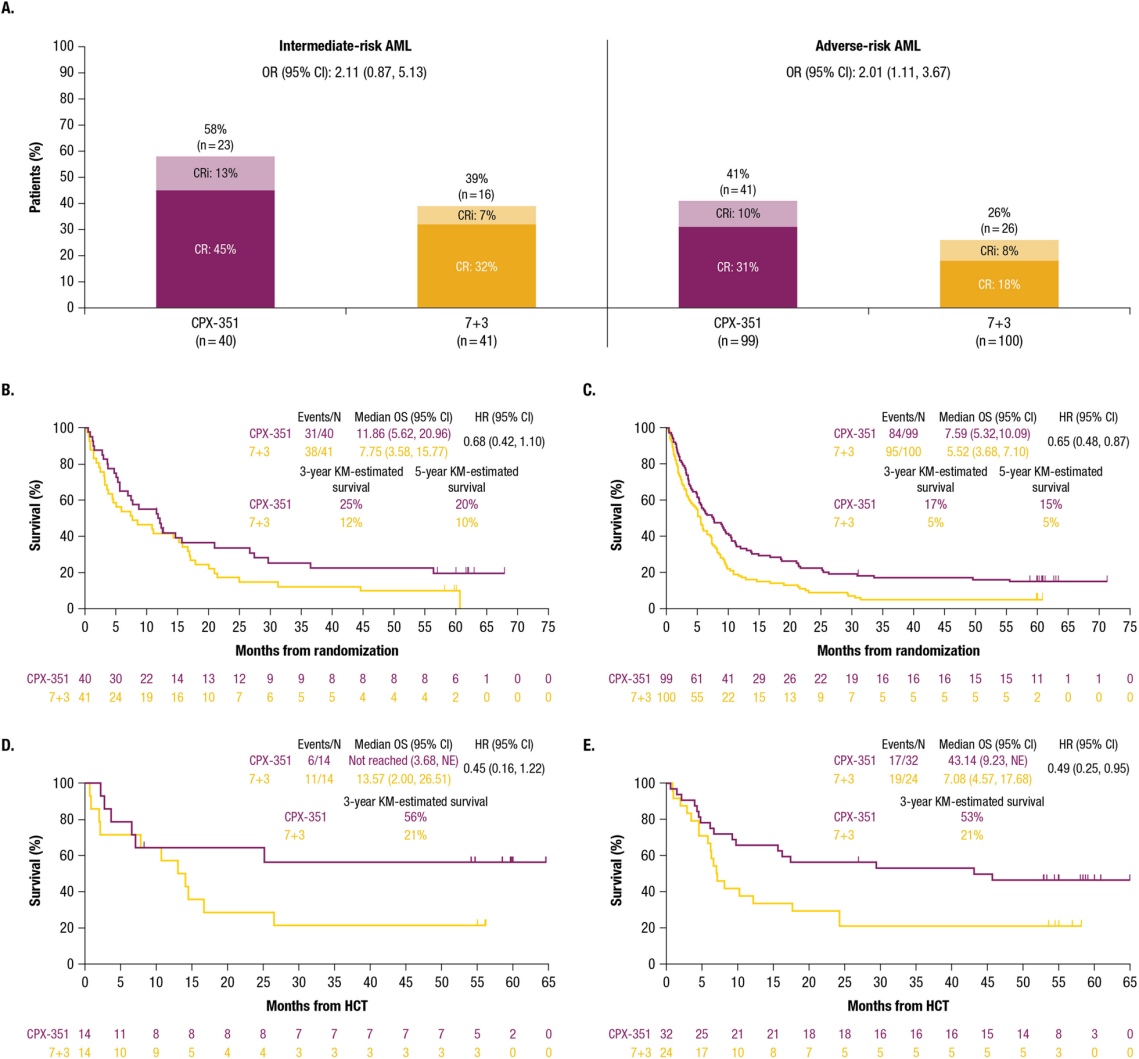
Efficacy Outcomes by ELN 2017 Risk Subgroup. **A** CR, CRi, and CR + CRi; **B** Kaplan-Meier OS for patients with intermediate-risk AML; **C** Kaplan-Meier OS for patients with adverse-risk AML; **D** Kaplan-Meier OS landmarked from the date of HCT for patients with intermediate-risk AML; **E** Kaplan-Meier OS landmarked from the date of HCT for patients with adverse-risk AML. *AML* acute myeloid leukemia; *CI* confidence interval; *CR* complete remission; *CRi* complete remission with incomplete neutrophil or platelet recovery; *ELN* European LeukemiaNet; *HCT* hematopoietic cell transplantation; *HR* hazard ratio; *KM* Kaplan-Meier; *NE* not estimable; *OR* odds ratio; *OS* overall survival

After a median follow-up of 60.65 months (interquartile range: 59.96, 62.09), median OS was longer in patients treated with CPX-351 versus 7 + 3 who had intermediate-risk or adverse-risk AML (Fig. 1B, C). Kaplan-Meier–estimated 5-year survival with CPX-351 and 7 + 3 was 20% and 10%, respectively, for patients with intermediate-risk AML and 15% and 5% for those with adverse-risk AML. Among patients with adverse-risk AML without *TP53* mutations, median OS was 9.6 months with CPX-351 (*n* = 75) versus 5.6 months with 7 + 3 (*n* = 69). Among those with adverse-risk AML and *TP53* mutations, median OS was 5.0 months with CPX-351 (*n* = 24) versus 5.1 months with 7 + 3 (*n* = 31; Additional file 1: Table S1).

Within the intermediate-risk subgroup, 14/40 (35%) patients treated with CPX-351 proceeded to HCT, including 10 patients in CR and 3 in CRi at the time of HCT, and 14/41 (34%) patients treated with 7 + 3 proceeded to HCT, including 8 patients in CR and 2 in CRi at the time of HCT. Median OS landmarked from the HCT date was not reached with CPX-351 versus 13.57 months with 7 + 3; the landmarked Kaplan-Meier–estimated 3-year survival was 56% versus 21% (Fig. 1D). Kaplan-Meier–estimated 5-year survival from the randomization date for transplanted patients was 56% versus 21%, respectively.

Within the adverse-risk subgroup, 32/99 (32%) patients treated with CPX-351 proceeded to HCT, including 16 patients in CR and 6 in CRi at the time of HCT, and 24/100 (24%) patients treated with 7 + 3 proceeded to HCT, including 10 patients in CR and 3 in CRi at the time of HCT. Median OS landmarked from the HCT date was 43.14 months with CPX-351 versus 7.08 months with 7 + 3; the landmarked Kaplan-Meier–estimated 3-year survival was 53% versus 21% (Fig. 1E). Kaplan-Meier–estimated 5-year survival from the randomization date for transplanted patients was 46% versus 21%, respectively. Among patients with adverse-risk AML without *TP53* mutations, median OS was not reached with CPX-351 (*n* = 28) versus 11.22 months with 7 + 3 (*n* = 14). Among those with adverse-risk AML and *TP53* mutations, median OS was 9.97 months with CPX-351 (*n* = 4) versus 6.41 months with 7 + 3 (*n* = 10; Additional file 1: Table S1).

Across risk groups and treatment arms, the most common adverse events were febrile neutropenia, gastrointestinal events, and peripheral edema (Additional file 1: Table S2). Serious febrile neutropenia occurred in 13% of patients with intermediate-risk AML treated with CPX-351 or 7 + 3 and in 4% and 2% of patients, respectively, with adverse-risk AML. Hematologic recovery times were longer with CPX-351 versus 7 + 3 among patients with intermediate-risk (23 vs 16 days) and adverse-risk (41 vs 26 days) AML who achieved remission (Additional file 1: Table S2). However, early mortality was lower with CPX-351 versus 7 + 3 among patients with intermediate-risk (Day 30: 5% vs 13%; Day 60: 13% vs 20%) and adverse-risk AML (Day 30: 6% vs 11%; Day 60: 16% vs 25%).

The estimated length (95% CI) of hospitalization per patient-year (normalized to median treatment duration) with CPX-351 and 7 + 3 was 184.5 days (175.3, 194.1) and 217.9 days (207.1, 229.4), respectively, in the intermediate-risk subgroup and 207.1 days (200.7, 213.8) and 260.9 days (251.9, 270.1) in the adverse-risk subgroup (Additional file 1: Table S3). The estimated number (95% CI) of platelet units administered per patient-year after CPX-351 and 7 + 3 was 81.0 (76.7, 85.5) and 61.1 (57.0, 65.4), respectively, in the intermediate-risk subgroup and 78.0 (75.2, 80.9) and 93.7 (89.9, 97.6) in the adverse-risk subgroup. The estimated number (95% CI) of red blood cell units administered after CPX-351 and 7 + 3 was 40.8 (37.8, 44.0) and 34.2 (31.2, 37.5) in the intermediate-risk subgroup and 39.5 (37.6, 41.6) and 48.8 (46.1, 51.6) in the adverse-risk subgroup (Additional file 1: Table S4).

This post hoc analysis of the final 5-year follow-up data from the phase 3 study demonstrated that CPX-351–treated patients had more frequent remission and longer median OS and post-HCT survival versus 7 + 3 in older adults with newly diagnosed, intermediate-risk or adverse-risk AML per the ELN 2017 risk criteria, with outcomes generally poorer among patients with adverse-risk AML, similar to prior analyses by NCCN risk groups [4]. The safety profile of CPX-351 among patients with intermediate-risk or adverse-risk AML was consistent with that of the overall study population [4, 6] and known safety profile of 7 + 3. Hematologic recovery times were longer with CPX-351 versus 7 + 3, which was expected based on longer bone marrow drug exposure following CPX-351, but also markedly longer in the adverse-risk subgroup (41 vs 26 days [15 days longer with CPX-351]) than the intermediate-risk subgroup (23 vs 16 days [7 days longer with CPX-351]). Despite prolonged myelosuppression with CPX-351, median OS was longer, early mortality was lower, and hospitalization length of stay per patient-year was shorter with CPX-351 versus 7 + 3 within the intermediate-risk or adverse-risk subgroups, with no consistent difference in transfusions.

CPX-351 comprises the same active drugs as the 7 + 3 regimen; however, the design of CPX-351 (liposomal encapsulation of daunorubicin and cytarabine in a synergistic 1:5 molar ratio) provides coordinated drug pharmacokinetics, prolonged drug exposure, maintenance of the synergistic drug ratio, and preferential uptake by AML cells in the bone marrow [7–10].

Although CPX-351 improved outcomes versus 7 + 3 for patients with adverse-risk AML, it is noteworthy that patients with *TP53* mutations responded poorly, regardless of therapy. NCCN guidelines recommend against the use of 7 + 3 and suggest alternative strategies or clinical trials should be explored in this patient subgroup [11]. Considering that the active compounds in CPX-351 are daunorubicin and cytarabine (same as for the 7 + 3 regimen), it is not unexpected that CPX-351 has limited benefit for patients with *TP53* mutations.

The longer median OS and post-HCT survival observed with CPX-351 versus 7 + 3 in this post hoc analysis of older adults with newly diagnosed, intermediate-risk or adverse-risk AML per the ELN 2017 risk criteria are consistent with observations reported for the overall study population [4, 6]. These results further support the use of CPX-351 in patients with newly diagnosed, therapy-related AML and AML-MRC.

## Supplementary Information

**Additional file 1**. Supplemental Data Appendix.

## Data Availability

All relevant data are provided within this manuscript and supporting files, or within the files for the previous study publications by Lancet JE, et al. *J Clin Oncol*. 2018;36(26):2684–2692 and Lancet JE, et al. *Lancet Haematol*. 2021;8(7):e481-e491.

## Abbreviations

AML: Acute myeloid leukemia
AML-MRC: AML with myelodysplasia-related changes
CI: Confidence interval
CMML: Chronic myelomonocytic leukemia
CPX-351: A dual-drug liposomal encapsulation of daunorubicin and cytarabine in a synergistic 1:5 molar ratio
CR: Complete remission
CRi: CR with incomplete neutrophil or platelet recovery
ECOG: Eastern Cooperative Oncology Group
ELN: European LeukemiaNet
HCT: Hematopoietic cell transplantation
HMA: Hypomethylating agent
HR: Hazard ratio
KM: Kaplan-Meier
NE: Not estimable
MDS: Myelodysplastic syndrome
NCCN: National Comprehensive Cancer Network
OR: Odds ratio
OS: Overall survival
t-AML: Therapy-related acute myeloid leukemia
WBC: White blood cell

## References

[1] Mayer LD, Tardi P, Louie AC (2019). CPX-351: a nanoscale liposomal co-formulation of daunorubicin and cytarabine with unique biodistribution and tumor cell uptake properties. Int J Nanomed.

[2] European Medicines Agency. Vyxeos liposomal (previously known as Vyxeos). https://www.ema.europa.eu/en/medicines/human/EPAR/vyxeos-liposomal. Accessed June 15, 2022.

[3] Vyxeos^®^ (daunorubicin and cytarabine) liposome for injection, for intravenous use [package insert]. Palo Alto, CA: Jazz Pharmaceuticals; 2021.

[4] Lancet JE, Uy GL, Cortes JE (2018). CPX-351 (cytarabine and daunorubicin) liposome for injection versus conventional cytarabine plus daunorubicin in older patients with newly diagnosed secondary acute myeloid leukemia. J Clin Oncol.

[5] Dohner H, Estey E, Grimwade D (2017). Diagnosis and management of AML in adults: 2017 ELN recommendations from an international expert panel. Blood.

[6] Lancet JE, Uy GL, Newell LF (2021). CPX-351 versus 7+3 cytarabine and daunorubicin chemotherapy in older adults with newly diagnosed high-risk or secondary acute myeloid leukaemia: 5-year results of a randomised, open-label, multicentre, phase 3 trial. Lancet Haematol.

[7] Feldman EJ, Kolitz JE, Trang JM (2012). Pharmacokinetics of CPX-351; a nano-scale liposomal fixed molar ratio formulation of cytarabine:daunorubicin, in patients with advanced leukemia. Leuk Res.

[8] Feldman EJ, Lancet JE, Kolitz JE (2011). First-in-man study of CPX-351: a liposomal carrier containing cytarabine and daunorubicin in a fixed 5:1 molar ratio for the treatment of relapsed and refractory acute myeloid leukemia. J Clin Oncol.

[9] Lim WS, Tardi PG, Dos Santos N (2010). Leukemia-selective uptake and cytotoxicity of CPX-351, a synergistic fixed-ratio cytarabine:daunorubicin formulation, in bone marrow xenografts. Leuk Res.

[10] Kim HP, Gerhard B, Harasym TO, Mayer LD, Hogge DE (2011). Liposomal encapsulation of a synergistic molar ratio of cytarabine and daunorubicin enhances selective toxicity for acute myeloid leukemia progenitors as compared to analogous normal hematopoietic cells. Exp Hematol.

[11] National Comprehensive Cancer Network. NCCN Clinical Practice Guidelines in Oncology (NCCN Guidelines): Acute Myeloid Leukemia. Version 2.2022. Plymouth Meeting, PA: National Comprehensive Cancer Network; 2022.

