# Supplementary material for: Efficacy and safety of CPX-351 versus 7 + 3 chemotherapy by European LeukemiaNet 2017 risk subgroups in older adults with newly diagnosed, high-risk/secondary AML: post hoc analysis of a randomized, phase 3 trial
Source: J Hematol Oncol. 2022 Oct 26;15:155. doi: 10.1186/s13045-022-01361-w (PMC9598030; doi:10.1186/s13045-022-01361-w)
Supplement: Supplementary file 1 — Additional file 1. Supplemental Data Appendix. [file 13045_2022_1361_MOESM1_ESM.docx]

**Supplemental Digital Content**

**Supplementary Table S1. Outcomes in Patients With Adverse-risk AML by *TP53* Mutation Status**

|  | **Wild-type *TP53*** | | ***TP53* mutation** | |
| --- | --- | --- | --- | --- |
|  | **CPX-351**  **(n=75)** | **7+3**  **(n=69)** | **CPX-351**  **(n=24)** | **7+3**  **(n=31)** |
| CR, n (%) | 23 (31) | 9 (13) | 8 (33) | 9 (29) |
| CR+CRi, n (%) | 33 (44) | 15 (22) | 8 (33) | 11 (35) |
| Median OS (95% CI), months | 9.56  (5.36, 11.33) | 5.55  (3.58, 7.52) | 4.98  (2.73, 7.59) | 5.13  (2.04, 7.36) |
| HCT, n (%) | 28 (37) | 14 (20) | 4 (17) | 10 (32) |
| Median OS landmarked from the date of HCT (95% CI), months | Not reached  (9.76, NE) | 11.22  (4.57, NE) | 9.97  (3.98, 45.70) | 6.41  (0.89, 8.15) |

Abbreviations: AML, acute myeloid leukemia; CI, confidence interval; CR, complete remission; CRi, complete remission with incomplete neutrophil or platelet recovery; HCT, hematopoietic cell transplantation; NE, not estimable; OS, overall survival.

**Supplementary Table S2. Safety Results by ELN 2017 Risk Subgroup**

|  | **Intermediate-risk AML** | | **Adverse-risk AML** | |
| --- | --- | --- | --- | --- |
|  | **CPX-351**  **(n=40)** | **7+3**  **(n=40)** | **CPX-351**  **(n=99)** | **7+3**  **(n=96)** |
| **Any TEAE (any grade),^a^ n (%)**  Febrile neutropenia  Nausea  Diarrhea  Peripheral edema  Headache  Epistaxis  Fatigue  Constipation  Cough  Decreased appetite  Vomiting  Chills  Mucosal inflammation | 40 (100)  34 (85)  19 (48)  18 (45)  18 (45)  15 (38)  15 (38)  14 (35)  13 (33)  12 (30)  11 (28)  11 (28)  10 (25)  10 (25) | 40 (100)  28 (70)  25 (63)  27 (68)  21 (53)  14 (35)  8 (20)  13 (33)  20 (50)  7 (18)  17 (43)  13 (33)  15 (38)  13 (33) | 99 (100)  62 (63)  47 (47)  44 (44)  37 (37)  34 (34)  34 (34)  31 (31)  44 (44)  31 (31)  31 (31)  24 (24)  22 (22)  14 (14) | 96 (100)  67 (70)  49 (51)  63 (66)  46 (48)  21 (22)  18 (19)  36 (38)  34 (35)  22 (23)  38 (40)  15 (16)  23 (24)  16 (17) |
| **Any serious AE,^b^ n (%)**  Febrile neutropenia  Sepsis  Respiratory failure  Ejection fraction decreased  Bacteremia  Pneumonia  Acute respiratory failure | 19 (48)  5 (13)  2 (5)  2 (5)  2 (5)  2 (5)  0  0 | 18 (45)  5 (13)  1 (3)  2 (5)  2 (5)  0  2 (5)  2 (5) | 44 (44)  4 (4)  6 (6)  5 (5)  4 (4)  2 (2)  5 (5)  2 (2) | 34 (35)  2 (2)  2 (2)  4 (4)  4 (4)  0  1 (1)  1 (1) |
| **Overall deaths, n (%)**  Death due to TEAE | 31 (78)  3 (8) | 37 (93)  7 (18) | 84 (85)  10 (10) | 91 (95)  14 (15) |
| **Early mortality at Day 30, n (%)** | 2 (5) | 5 (13) | 6 (6) | 11 (11) |
| **Early mortality at Day 60, n (%)** | 5 (13) | 8 (20) | 16 (16) | 24 (25) |
| **Platelet recovery in patients with CR or CRi**  n  Median time to platelets ≥50,000/μL (range), days | 23  42.0 (28, 76) | 16  29.0 (21, 49) | 41  35.0 (14, 106) | 26  28.0 (21, 144) |
| **ANC recovery in patients with CR or CRi**  n  Median time to neutrophils ≥500/μL (range), days | 23  36.0 (28, 50) | 16  29.5 (21, 42) | 41  35.0 (21, 78) | 26  28.0 (20, 38) |

AE, adverse event; AML, acute myeloid leukemia; ANC, absolute neutrophil count; CR, complete remission; CRi, complete remission with incomplete neutrophil or platelet recovery; ELN, European LeukemiaNet; TEAE, treatment-emergent adverse event.

^a^List of individual TEAEs includes all TEAEs occurring in ≥30% of patients in any subgroup.

^b^List of individual serious AEs includes all serious AEs occurring in ≥5% of patients in any subgroup.

**Supplementary Table S3. Hospital Length of Stay by ELN 2017 Risk Subgroup**

|  | **Intermediate-risk AML** | | **Adverse-risk AML** | |  |
| --- | --- | --- | --- | --- | --- |
|  | **CPX-351**  **(n=40)** | **7+3**  **(n=40)** | **CPX-351**  **(n=99)** | **7+3**  **(n=96)** | |
| **All patients, n**  Median total days in hospital during treatment (range)  Estimated days in hospital PPY during treatment (95% CI)  Median total days in ICU during treatment (range)  Estimated days in ICU PPY during treatment (95% CI) | 40  39.5 (3, 67)  184.5 (175.3, 194.1)  0 (0, 11)  3.1 (2.1, 4.6) | 40  35 (10, 83)  217.9 (207.1, 229.4)  0 (0, 17)  9.1 (7.0, 11.6) | 99  38 (8, 110)  207.1 (200.7, 213.8)  0 (0, 29)  5.9 (4.9, 7.1) | 96  30 (2, 77)  260.9 (251.9, 270.1)  0 (0, 16)  10.3 (8.6, 12.2) | |
| **Patients with CR, n**  Median total days in hospital during treatment (range)  Estimated days in hospital PPY during treatment (95% CI)  Median total days in ICU during treatment (range)  Estimated days in ICU PPY during treatment (95% CI) | 18  43.5 (9, 64)  152.6 (142.0, 163.9)  0 (0, 11)  2.2 (1.2, 4.1) | 13  48 (29, 83)  165.5 (153.2, 178.7)  0 (0, 17)  6.1 (4.1, 9.2) | 31  37 (15, 61)  135.4 (128.0, 143.3)  0 (0, 3)  0.7 (0.3, 1.5) | 18  41.5 (25, 77)  198.2 (184.9, 212.4)  0 (0, 11)  9.7 (7.1, 13.3) | |
| **Patients with CRi, n**  Median total days in hospital during treatment (range)  Estimated days in hospital PPY during treatment (95% CI)  Median total days in ICU during treatment (range)  Estimated days in ICU PPY during treatment (95% CI) | 5  45 (33, 67)  185.4 (163.2, 210.5)  0 (0, 0)  – | 3  42 (39, 53)  271.9 (229.6, 322.1)  0 (0, 7)  14.2 (6.8, 29.8) | 10  52 (33, 110)  248.8 (229.1, 270.1)  0 (0, 7)  5.7 (3.3, 9.8) | 8  45 (29, 67)  215.5 (194.4, 238.8)  0 (0, 0)  – | |

AML, acute myeloid leukemia; CR, complete remission; CRi, complete remission with incomplete neutrophil or platelet recovery; CI, confidence interval; ELN, European LeukemiaNet; ICU, intensive care unit; PPY, per patient-year.

To normalize the data to address differences in the length of the treatment period between arms (overall, median of 62 days with CPX-351 vs 41 days with 7+3),^4^ estimated incidence rates PPY and associated 95% CIs were calculated based on exact Poisson distributions.

**Supplementary Table S4. Transfusions Administered by ELN 2017 Risk Subgroup**

|  | **Intermediate-risk AML** | | **Adverse-risk AML** | |
| --- | --- | --- | --- | --- |
|  | **CPX-351**  **(n=40)** | **7+3**  **(n=40)** | **CPX-351**  **(n=99)** | **7+3**  **(n=96)** |
| **All patients, n**  Median bags of platelets administered (range)  Estimated bags of platelets administered PPY (95% CI)  Median bags of PRBCs administered (range)  Estimated bags of PRBCs administered PPY (95% CI) | 40  20.0 (4.0, 213.9)  81.0 (76.7, 85.5)  14.2 (0.0, 40.0)  40.8 (37.8, 44.0) | 40  15.0 (0.0, 69.4)  61.1 (57.0, 65.4)  12.1 (2.0, 30.0)  34.2 (31.2, 37.5) | 99  15.0 (1.0, 230.0)  78.0 (75.2, 80.9)  14.0 (0.0, 40.3)  39.5 (37.6, 41.6) | 96  10.0 (0.0, 192.7)  93.7 (89.9, 97.6)  10.0 (0.0, 41.0)  48.8 (46.1, 51.6) |
| **Patients with CR, n**  Median bags of platelets administered (range)  Estimated bags of platelets administered PPY (95% CI)  Median bags of PRBCs administered (range)  Estimated bags of PRBCs administered PPY (95% CI) | 18  18.5 (4.0, 213.9)  72.2 (67.1, 77.8)  15.4 (4.8, 31.0)  29.1 (25.9, 32.7) | 13  9.0 (2.0, 44.0)  21.9 (18.8, 25.4)  8.0 (2.0, 30.0)  18.6 (15.8, 21.9) | 31  15.0 (2.0, 144.7)  45.2 (42.2, 48.4)  14.0 (3.0, 30.7)  27.5 (25.1, 30.0) | 18  8.5 (2.0, 121.0)  44.4 (40.0, 49.2)  11.4 (5.8, 24.3)  25.6 (22.3, 29.3) |
| **Patients with CRi, n**  Median bags of platelets administered (range)  Estimated bags of platelets administered PPY (95% CI)  Median bags of PRBCs administered (range)  Estimated bags of PRBCs administered PPY (95% CI) | 5  31.0 (11.0, 68.0)  74.8 (64.9, 86.2)  19.0 (8.7, 31.9)  38.9 (32.0, 47.4) | 3  25.0 (20.0, 53.0)  99.4 (81.6, 121.2)  13.0 (9.9, 16.0)  39.5 (28.8, 54.1) | 10  18.0 (10.0, 31.0)  42.4 (36.8, 48.8)  15.5 (12.0, 33.3)  37.8 (32.6, 43.9) | 8  11.5 (4.0, 192.7)  87.5 (78.1, 98.1)  18.0 (9.2, 24.5)  41.6 (35.3, 49.1) |

AML, acute myeloid leukemia; CR, complete remission; CRi, complete remission with incomplete neutrophil or platelet recovery; CI, confidence interval; ELN, European LeukemiaNet; PPY, per patient-year; PRBC, packed red blood cell.

To normalize the data to address differences in the length of the treatment period between arms (overall, median of 62 days with CPX-351 vs 41 days with 7+3),^4^ estimated incidence rates PPY and associated 95% CIs were calculated based on exact Poisson distributions.
